# Outlines of the Course of Qualitative Analyses Followed in the Giessen Laboratory

**Published:** 1846-07

**Authors:** 


Outlines of the Course of Qualitative Analyses followed in the Giessen Laboratory. By *Henry Will*, Ph. D., Professor Extraordinary of Chemistry in the University of Giessen. With a Preface by Baron *Liebig*. London. 1846.

Translations of the works of German chemists into English have lately been abundant, and it would be unfair to deny that they have been useful to the student; and the present work, though somewhat wanting in details, forms no exception to the general character of the class. As a translation we have no doubt that it is a correct one, but this is not all that is wanted or expected by the pupil; the original should have been accompanied with notes by the translator to have rendered it extensively useful: there are besides other deficiencies—for example, though we have abundant formulæ, there is no list of equivalents appended to the book, so that we have to guess at their value. Baron Liebeg, in the Preface, has stated that "the present work is designed for the use of the laboratory; consequently everything, which does not immediately refer to the processes of analysis, is very properly excluded." Let us admit the accuracy of this statement, and yet the praise is of a truly negative description. It would have been more to the purpose, if the Baron could have assured us that it does contain all or even a large portion of what *does* immediately refer to the subject treated of.

We shall offer a few observations on parts of this work taken entirely at random, and, if the translation be faithful, the original contains statements which surprise us as coming from so well known and justly appreciated an authority as Dr. Will. For example, it is mentioned, with respect to oxide of manganese, that "most of the salts of this oxide dissolve in water, yielding pale rose-coloured solutions, and all of them are soluble in hydrochloric acid." Now, though we have seen slightly reddish-coloured solutions of manganesian salts, we believe the colour to have arisen entirely from some of the metal being in the state of acid. Of this we are at any rate perfectly sure, that we possess solutions of protosalts of manganese which are perfectly colourless, and therefore to state that a pale rose-colour is characteristic of them is utterly fallacious.

Under the head of "Acids of Nitrogen," we have first nitric acid represented by HO, $NO^5$, and it is stated that "pure hydrate of nitric acid is a colourless liquid, diffusing white fumes of a pungent odour, when in contact with the atmosphere." Now the strongest nitric acid obtainable is a sesqui-hydrate, and ought therefore to be represented by 3HO, $2NO^5$.

It is moreover stated that, "with the exception of binoxide of tin, peroxide of antimony, [tellurous and tungstic acid,] all oxides are soluble in an excess of nitric acid"; this is surely a mis-statement, for binoxide of manganese requires the presence of a deoxidizing agent, as sugar, to become soluble in nitric acid; and binoxide of lead is also insoluble in it, and red oxide of lead is decomposed by its action. It would surely, therefore, disappoint and surprise the student to find no action whatever occurring in the two first cases, and that, in the third, he obtained a brown powder insoluble in the acid.

The author states that, "when ignited, carbonates lose their carbonic acid, with the exception of the carbonates of the alkalies and some alkaline earths (baryta and strontia)." Now carbonate of baryta we know, and that of strontia we believe, to be decomposed by heat.

Some other statements have struck us as requiring correction, and these, we have no doubt, that the translator will discover to be erroneous; and by the time that a new edition of the work is required, we trust that the requisite attention will be paid to the alterations requisite to render it perfectly worthy of the confidence of the chemical student.

